# Platinum-Quality Mitogenome Haplotypes from United States Populations

**DOI:** 10.3390/genes11111290

**Published:** 2020-10-29

**Authors:** Cassandra R. Taylor, Kevin M. Kiesler, Kimberly Sturk-Andreaggi, Joseph D. Ring, Walther Parson, Moses Schanfield, Peter M. Vallone, Charla Marshall

**Affiliations:** 1 Armed Forces Medical Examiner System’s Armed Forces DNA Identification Laboratory (AFMES-AFDIL), Dover Air Force Base, DE 19002, USA; cassandra.r.taylor7.ctr@mail.mil (C.R.T.); kimberly.s.andreaggi.ctr@mail.mil (K.S.-A.); joseph.d.ring2.ctr@mail.mil (J.D.R.); 2 SNA International, LLC; Alexandria, VA 22314, USA; 3 National Institute of Standards and Technology (NIST), Gaithersburg, MD 20899, USA; kevin.kiesler@nist.gov (K.M.K.); peter.vallone@nist.gov (P.M.V.); 4 Institute of Legal Medicine, Medical University of Innsbruck, Innsbruck 6020, Austria; walther.parson@i-med.ac.at; 5 Forensic Science Program, The Pennsylvania State University, State College, PA 16801, USA; 6 Department of Forensic Sciences, The George Washington University, Washington, DC 20007, USA; mschanfi@gwu.edu

**Keywords:** mtDNA, mitogenome, next-generation sequencing, haplotype, haplogroup, population statistics

## Abstract

A total of 1327 platinum-quality mitochondrial DNA haplotypes from United States (U.S.) populations were generated using a robust, semi-automated next-generation sequencing (NGS) workflow with rigorous quality control (QC). The laboratory workflow involved long-range PCR to minimize the co-amplification of nuclear mitochondrial DNA segments (NUMTs), PCR-free library preparation to reduce amplification bias, and high-coverage Illumina MiSeq sequencing to produce an average per-sample read depth of 1000 × for low-frequency (5%) variant detection. Point heteroplasmies below 10% frequency were confirmed through replicate amplification, and length heteroplasmy was quantitatively assessed using a custom read count analysis tool. Data analysis involved a redundant, dual-analyst review to minimize errors in haplotype reporting with additional QC checks performed by EMPOP. Applying these methods, eight sample sets were processed from five U.S. metapopulations (African American, Caucasian, Hispanic, Asian American, and Native American) corresponding to self-reported identity at the time of sample collection. Population analyses (e.g., haplotype frequencies, random match probabilities, and genetic distance estimates) were performed to evaluate the eight datasets, with over 95% of haplotypes unique per dataset. The platinum-quality mitogenome haplotypes presented in this study will enable forensic statistical calculations and thereby support the usage of mitogenome sequencing in forensic laboratories.

## 1. Introduction

Advances in next-generation sequencing (NGS) technologies allow for efficient whole-mitochondrial-genome (mitogenome) sequence analysis of high-quality and degraded DNA samples [1,2,3,4]. NGS generates large amounts of data per sample and high read depth, which allows for increased sensitivity [3,5,6]. The use of automated processing in NGS, especially for library preparation, reduces hands-on time and thus decreases the risk of human error (e.g., contamination and sample switches) [7]. Additionally, the automated nature of NGS data analysis minimizes bias in data interpretation. Homopolymer stretches can be analyzed quantitatively to determine the major molecule within the sequence data, which is an improvement over the visual method applied to the capillary electrophoresis data produced from Sanger sequencing [8,9,10]. The identification and removal of nuclear mitochondrial DNA segments (NUMTs) is also possible in NGS analysis using bioinformatic tools to investigate phasing of variants along parsed sequence reads and to perform multiple reference sequence alignment [11,12]. Automatic haplogrouping of complete mitogenome sequences is an additional quality control (QC) measure that can be used to flag unusual or phylogenetically implausible haplotypes arising from processing errors. Hence, NGS allows for more thorough QC of mitochondrial DNA (mtDNA) sequences than Sanger sequencing.

MtDNA data are numerous in the published literature, but sufficient quality-controlled mitogenome data for haplotype frequency estimations are lacking. The European DNA Profiling Group (EDNAP) mtDNA Population Database (EMPOP) is the preferred mtDNA haplotype database of the forensic community because it uses a robust QC pipeline to minimize the inclusion of error-ridden haplotypes [13,14]. At the time of writing, EMPOP contains only 4289 forensic-quality entire mitogenome profiles for haplotype frequency estimation. With the support of EMPOP and the Institut für Gerichtliche Medizin (GMI), the Armed Forces Medical Examiner System’s Armed Forces DNA Identification Laboratory (AFMES-AFDIL) was awarded a National Institute of Justice (NIJ) grant to augment the EMPOP database with mitogenome haplotypes from 4000 United States (U.S.) and 1000 global population samples. Many of the samples were previously sequenced in the control region (CR) with Sanger sequencing [15,16,17,18], allowing for comparison between the NGS and Sanger CR data and thus additional QC. In this report, the first completed datasets from the NIJ grant project are presented. As part of this effort, mitogenomes produced by the Applied Genetics group at the National Institute of Standards and Technology (NIST) were provided to the AFMES-AFDIL for analysis and inclusion in this study.

Over 1300 mtDNA profiles were generated using a thoroughly tested NGS processing method with automated data analysis and rigorous QC measures [19,20]. This method utilizes two-amplicon long-range PCR amplification, the KAPA HyperPlus Library Preparation Kit (Roche Sequencing, Waltham, MA, USA), and sequencing on the Illumina MiSeq (Illumina, San Diego, CA, USA). The two-amplicon approach minimizes the potential for co-amplification of nuclear mtDNA segments (NUMTs) that can complicate data analysis [11,21,22]. The mitigation of NUMT interference with long-range enrichment allows for the use of a lower variant frequency threshold than short amplicon and whole-genome sequencing methods [11,23]. The library preparation method used in this study, KAPA HyperPlus, was shown to produce even coverage across the mitogenome to maximize sequencing efficiency [19]. Furthermore, this library preparation method can accommodate a wide range of DNA input and does not require library amplification. KAPA HyperPlus therefore minimizes amplicon dilution prior to library preparation and eliminates the potential for PCR bias introduced during the library procedure [19]. The Illumina MiSeq sequencing performed herein is advantageous due to its higher throughput and robust performance in homopolymer regions compared to pH-based sequencing [9,24]. Using an automated laboratory and analysis pipeline with a redundant data review, low-frequency variant confirmation through independent replication, and external EMPOP QC, ‘platinum-quality’ mitogenome haplotypes suitable for forensic use are provided.

## 2. Materials and Methods

### 2.1. Sample Description

Eight anonymized sample sets were utilized to generate mitogenomes of unrelated individuals from five U.S. metapopulations: Caucasian, African American, Hispanic, Asian American, and Native American (Table 1). Sample classification was based on self-reported metapopulation at the time of sample collection. No attempt to modernize population naming was performed by the authors, as it was not possible to trace samples back to the original donors in order to update the self-reported classification based on modern terms. However, it is acknowledged that more inclusive terminology would be preferable (e.g., LatinX instead of Hispanic). The samples used in this study were obtained from the following three sources: the Analytical Genetic Testing Center located in Colorado (AGTC-CO), NIST, and the Department of Defense Serum Repository (DoDSR). For ease of naming, each sample set was assigned a four-letter acronym; the first two letters reference the sample source (CO for AGTC-CO, NT for NIST, and DS for DoDSR) and the last two letters reference the metapopulation (AF for African American, CN for Caucasian, HS for Hispanic, AS for Asian American, and NA for Native American). This study was reviewed and approved by the Institutional Review Board Office (IRBO) under the U.S. Army Medical Research and Material Command’s Office of Research Protections (IRBO log number M-10185); it was determined to be research not involving human subjects because the samples were anonymous and no personally identifiable information was obtained. The NIST Research Protections Office reviewed and approved the use of the NIST population samples for this work. 

The first three sample sets (COAF, COCN, and COHS) included 354 samples from the AGTC-CO paternity cases and were stored at the Department of Forensic Sciences at The George Washington University (Washington, DC). A majority of these specimens were collected from individuals living in Colorado, though some samples may have originated from individuals in other U.S. states. The next three sample sets (NTAF, NTCN, and NTHS) included 659 samples from NIST, collected from several blood banks within the U.S. [25]. Two additional sample sets (DSAS and DSNA), included 350 samples from the DoDSR, and were collected from U.S. service members who self-identified as Asian American or Native American. The geographic location associated with each DoDSR sample was the home state of record. Of note, seven individuals in the DSAS sample set listed U.S. territories in the Pacific as the home state of record (Guam *n* = 5; American Samoa *n* = 2). DNA was extracted from the samples using either the QIAamp 96 DNA Blood Kit (QIAGEN, Hilden, Germany) or a salting out method [26,27]. CR data previously generated with Sanger sequencing were available for the AGTC-CO and most of the NIST samples (see Supplemental File 1 for CR haplotypes and Table S1 for CR EMPOP/GenBank accession numbers) [15,16,17,18,25].

### 2.2. Two-Amplicon Mitogenome Enrichment, Library Preparation, and Sequencing

Mitogenome sequence data were produced from the three primary sample sources (AGTC-CO, NIST, and DoDSR) using slightly different NGS processing techniques, as shown in Table 2. The AGTC-CO and DoDSR samples were processed at the AFMES-AFDIL, whereas the NIST samples were processed at NIST. The AGTC-CO and NIST samples were processed manually, and the DoDSR samples were processed using an automated post-amplification procedure performed on a Hamilton STARplus (Hamilton Company, Reno, NV, USA). Other slight modifications to the overall laboratory procedure included: amplification input and purification, library input and reaction volume, and MiSeq sequencing parameters. The mitogenome of each sample was enriched using long-range PCR amplification of two overlapping targets of ~8.5 kb (amplicon A (2A): nucleotide positions (nps) 2499–10,837; amplicon B (2B): nps2669–10,672), following the conditions described in Peck et al [20]. Primer sequences can be found in Table S2. The resulting amplicons were quantified with either the dsDNA 920 Reagent Kit (75 bp–15,000 bp) or the DNF-492 Large Fragment Kit (50 bp–20,000 bp) (Agilent Technologies, Santa Clara, CA, USA) on the Fragment Analyzer System (Agilent Technologies). Based on amplicon yield, 2A and 2B amplicons of each sample were pooled together. For the AGTC-CO samples, 2A and 2B were combined in equal volumes (12.5 µL), with the exception of 94 samples in which 11 µL of 2A was combined with 14 µL of 2B. This was performed to streamline processing by combining 2A and 2B amplicons based on the average concentration for the sample plate. This amplicon pooling method was also utilized for the samples sequenced at the NIST. For the DoDSR samples, 2A and 2B were normalized individually by concentration. The AGTC-CO and DoDSR samples were purified with AMPure XP (Beckman Coulter Life Sciences, Indianapolis, IN, USA) prior to library preparation (Table 2). The amplicon pools were converted to Illumina libraries with the KAPA HyperPlus Library Preparation Kit, using either a full-reaction method or the half-reaction method described by Ring et al. [19]. Library amplification was not performed in order to minimize PCR bias in the resulting sequence data [28]. The quality of the purified libraries was assessed with the DNF-474 High-Sensitivity NGS Fragment Analysis Kit (1 bp–6000 bp) (Agilent Technologies) on the Fragment Analyzer System. The libraries were then pooled together in equal volumes and sequenced on MiSeq FGx Forensic Genomics Systems (Verogen, San Diego, CA, USA). Single-end sequencing was performed for the AGTC-CO sample sets with the 150 cycle v3 MiSeq Reagent Kit (Illumina). Paired-end data were generated for the DoDSR and NIST sample sets using the 600 cycle v3 MiSeq Reagent Kit (Illumina).

### 2.3. Data Analysis

The CLC Genomics Workbench version 7.5.1 (QIAGEN) was used to import the demultiplexed FASTQ files generated by the MiSeq Reporter (Illumina) and analyze each sample with workflows customized for the HyperPlus data (Supplemental File 2). Before the reads are aligned to the revised Cambridge Reference Sequence (rCRS) [29,30], the workflow trimmed 20 nucleotides off of the 5′ end of each read and discarded reads less than 40 bp in length. Additionally, 20 nucleotides were trimmed off the 3′ end of each paired read of the DoDSR and NIST data. For read alignment, a length fraction of 1.0 and a similarity fraction of 0.8 were used. The low-frequency variant detection tool was applied for variant calling with a minimum read depth threshold of 100 × and a 5% variant frequency (VF) threshold. A base quality filter (neighborhood radius = 5, minimum central quality = 30, and minimum neighborhood quality = 30) and a 1% read direction (forward/reverse balance) filter were also used. Point heteroplasmy (PHP), in which two or more bases were observed above 5% frequency at a single nucleotide position, was indicated by using the appropriate International Union of Pure and Applied Chemistry (IUPAC) code. The AFDIL-QIAGEN mtDNA Expert (AQME) tool [8] was used to produce a reportable forensic mtDNA profile and predict the haplogroup based on PhyloTree build 17 [31,32].

**Figure 1:**
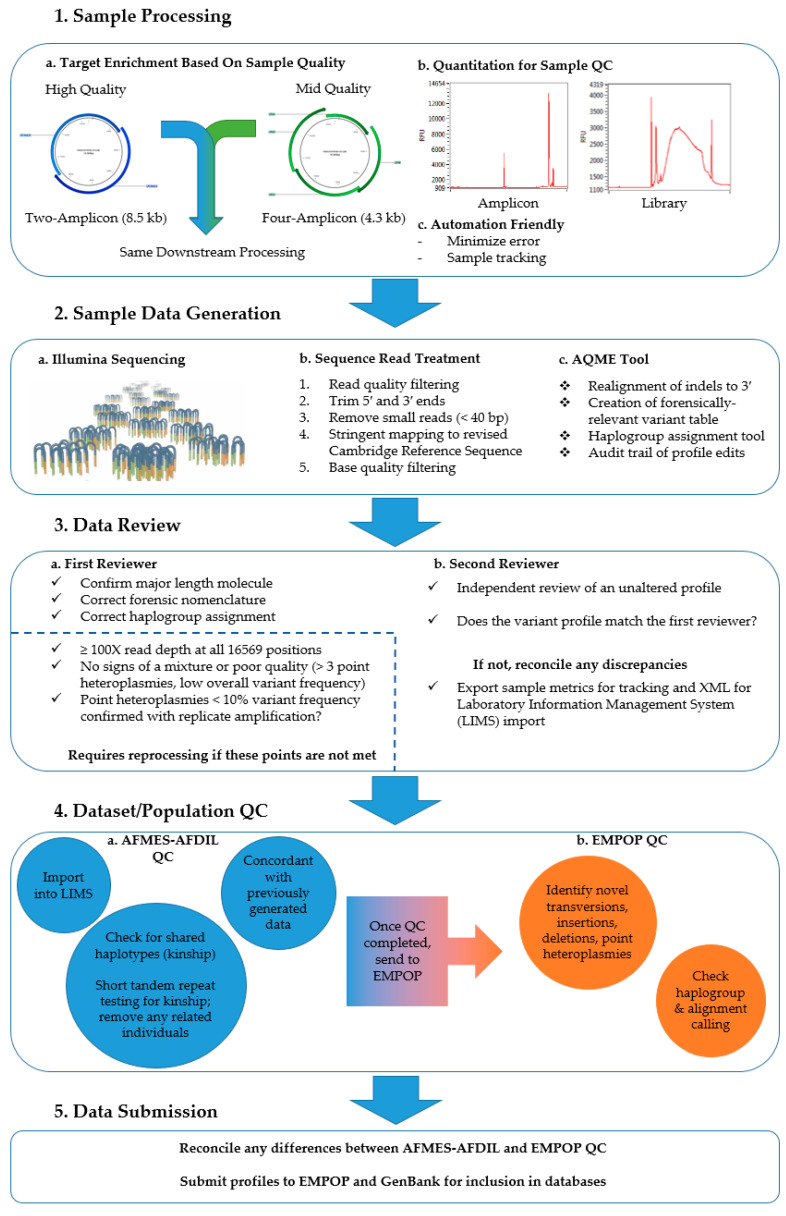
Summary of methods and quality control (QC) process for the generation of platinum-quality mitochondrial genomes. AFMES-AFDIL = Armed Forces Medical Examiner System’s Armed Forces DNA Identification Laboratory; EMPOP = The European DNA Profiling Group (EDNAP) mtDNA Population Database.

The CLC workflow generated two variant tables for each sample, allowing for blind review by two different analysts. Following forensic convention [14], the major (or dominant) length molecule in the profile was represented. Therefore, the workflow included a step to filter indels in regions that commonly exhibit length heteroplasmy (i.e., nps 303–315, 452–463, 513–524, 565–573, 956–966, 5891–5899, 8270–8289, 12,418–12,425, and 16,180–16,193). As variant calling in these regions can be complicated by alignment, the major length molecule was further confirmed using the read count analysis tool included in AQME [9]. In short, the read count analysis tool assessed the reads that extended across the entirety of each length heteroplasmy region, grouping them by sequence motif. Those sequence motifs with five or more reads were listed in the output. The motif of the sequence with the most reads (i.e. the major length molecule) was reported. Since length heteroplasmy can confound variant calling, special attention was paid to the sequence motifs listed in the read count analysis output in order to determine the presence of PHP in these regions. Any necessary profile edits, such as major length molecule adjustments described above or modifications required for adherence with forensic nomenclature [14,33], were made manually and tracked automatically in the audit trail for each sample [8]. The two profiles generated by the two analysts were compared by the secondary reviewer. If there were differences in the profiles, the profile with errors was sent back to the analyst for correction and then for re-approval by the secondary reviewer (Figure 1). Once the two analyses were concordant, the following outputs were exported from the CLC Genomics Workbench: a sample history report; an Excel file containing variant, coverage, mapping, and haplogroup information; and a CODIS-formatted XML file. Analysis metrics, such as read depth, major base frequency, and forward/reverse balance, were electronically collected and transferred to a Microsoft Access database (Microsoft Corp., Redmond, WA, USA) for further analysis. The mitogenome haplotype and interpretation range were electronically imported into Laboratory Information Systems Applications (LISA; Future Technologies Inc., Fairfax, VA, USA) via the XML files exported from CLC.

### 2.4. Sample Reprocessing 

Libraries resulting in partial profiles with nucleotide positions under the 100 × read depth threshold were re-pooled and re-sequenced at lower sample multiplexing to achieve a higher read depth per sample. Samples with multiple PHPs and/or reduced major base frequencies were flagged as possible mixtures. These samples were re-amplified and carried through the two-amplicon processing method. If samples were determined to be degraded (DNA fragments < 8500 bp) based on failure of one or both amplicons, an alternate enrichment approach was utilized with four smaller amplicons of approximately 4300 bp each [34] (Table S2). The samples that were processed with the four-amplicon enrichment were analyzed at a 2% VF threshold in addition to the 5% VF threshold in order to check for NUMT interference in the data.

### 2.5. Quality Control

After processing and data analysis were complete, all profiles were subjected to a thorough QC review as summarized in Figure 1. The first step was to ensure that the observed PHPs were authentic. To accomplish this, all PHPs with a minor base frequency < 10% were confirmed through independent amplification and downstream processing. Samples with PHPs were assessed critically to rule out the possibility that the PHP was due to a mixture. Mixtures were defined as haplotypes with reduced variant frequency overall and the presence of PHPs at sites of haplogroup-diagnostic variants, and they were excluded from the final datasets. All confirmed PHPs were visualized using the circlize package v0.4.10 in R version 4.0.2 [35,36] to show their distribution and frequency across the mitogenome.

Nuclear DNA testing with PowerPlex Fusion, PowerPlex 16, (Promega, Madison, WI, USA) or the ForenSeq DNA Signature Prep Kit (Verogen) was performed on samples with shared mitogenome haplotypes (excluding PHP and length heteroplasmy differences) for kinship assessment [37]. The PowerPlex 16 and PowerPlex Fusion data were analyzed using GeneMapper ID-X or previous versions (ThermoFisher Scientific, Waltham, MA, USA) and the ForenSeq data were analyzed using the ForenSeq Universal Analysis (UAS) Software (Verogen). Possible relatedness between samples with shared mitogenome haplotypes was assessed using Familias 3.0 [38,39] with allele frequency data for each respective metapopulation [40,41]. If two samples were determined to be from first-degree relatives (i.e., parent-child or siblings), only one of the mitogenome haplotypes from the first-degree relatives was included in the database. Additionally, if short tandem repeat (STR) testing revealed two samples with the same STR profile, either due to sample duplication or the presence of identical twins in the sample set, one was removed from the database. Nuclear DNA testing was also performed to evaluate the possibility of a sample mixture when needed.

During QC, the NGS data were compared to the previously generated CR Sanger data that were available for the AGTC-CO and most NIST samples (Supplemental File 1). This was performed for concordance and to identify any possible errors in processing. The available CR data were also used for PHP comparison. Only high-quality mitogenome data that passed the internal QC process were sent to EMPOP for review and further QC using tools detailed in Zimmermann et al. [42] and Huber et al. [43]. Finalized mitogenome haplotypes were submitted for inclusion in the EMPOP database [13] and the previous CR data for those samples were removed. Mitogenome haplotypes will also be submitted to GenBank [44]. The mtDNA haplogroup confirmed by EMPOP with SAM 2 [43] was used to predict mitochondrial ancestry on a continental level (i.e. African, Asian, European, and Native American) [45].

### 2.6. Population-Level Analyses

LISA was used to run pairwise comparisons of mitogenome haplotypes within each metapopulation to determine the number of shared and unique haplotypes. These pairwise comparisons were completed for both the CR (nps 16,024–16,569, 1–576) and the whole mitogenome (nps 1–16,569). Pairwise comparisons were performed with and without consideration of PHPs as differences. Length variants were excluded when running the pairwise comparisons in accordance with current forensic guidelines for haplotype comparison and database queries [14]. Specifically, insertions at these common positions of length heteroplasmy were ignored: 16,193, 309, 315, 455, 463, 573, 960, 5899, 8276, and 8285.

The pairwise comparisons were used to determine the empirical random match probability (RMP) and haplotype diversity for each sample set. Genetic distance between datasets was estimated from haplotypic data by computing pairwise Fst values in Arlequin v3.5 [46] using a Kimura 2-parameter model. The pairwise Fst matrix was used for a principal coordinate analysis (PCoA), performed in GenoDive version 3.0 [47]. Results were visualized in a 3-dimensional PCoA plot using the scatterplot3d package v0.3-14 in R version 4.0.2 [35,48].

## 3. Results

### 3.1. Sample Quality Metrics

The first-pass success rate with two-amplicon processing was >85% for all sample sets. An alternate four-amplicon enrichment approach was used on 29 samples (10 NIST and 19 DoDSR) in which the two-amplicon approach was unsuccessful. This approach was unsuccessful in five (17.2%) cases. A total of 32 samples (19 AGTC-CO, 3 NIST, and 10 DoDSR) were excluded from the finalized datasets after reprocessing attempts resulted in failed data (18) or mixed profiles (14) (Table 3). Additionally, four sets of duplicate/related samples were identified (discussed in Section 3.3), resulting in the exclusion of one sample in each set.

Platinum-quality mitogenome haplotypes were generated for 1327 individuals, representing eight datasets from five U.S. metapopulations (Supplemental Files 3 and 4). EMPOP accession numbers are provided in Table S1. All samples met or exceeded the minimum read depth threshold (100×) for the entire mitogenome with 1802× average read depth. Additionally, these data yielded average major base frequencies greater than 99% when heteroplasmy (both point and length variants) were excluded (Table 4). For detailed analysis metrics, see Table S3.

### 3.2. NUMT Interference in Four-Amplicon Data

When analyzed with a 2% VF threshold, NUMT interference was seen in the sequencing data of three NIST samples that had been amplified with the four-amplicon approach. In all three samples, the NUMTs were observed in the 4C amplicon (nps 6636–11,428; Table S2), which has primers homologous with portions of chromosome 5 [34]. The NUMTs were observed at 2–3%, below the minor variant detection threshold of 5%, and therefore did not affect the finalized data.

### 3.3. STR and Kinship Analyses of Samples with Shared Haplotypes

STR testing was performed on 126 samples (43 AGTC-CO, 63 NIST, and 20 DoDSR) with shared haplotypes within each dataset. No first-degree relatives were identified. However, two sets (one in NTCN and one in NTHS) of second- or third-degree relatives were identified (from autosomal STR and single nucleotide polymorphism (SNP) data; Table S4). One sample from each of these two sets of relatives was removed from the final datasets. It is noteworthy that both sets of relatives originated from samples with sequential numbering, indicating that the samples were likely collected consecutively. Two sets of duplicate samples, both in COAF, were identified through STR testing, and thus one of each duplicate was removed. Altogether, the kinship testing portion of the QC process helped to identify four samples that required exclusion from the database. These analyses did not include samples that were excluded from these datasets based on previous testing (e.g., during CR data generation).

### 3.4. CR Sanger Concordance

Excluding differences arising from low-level heteroplasmy, all mitogenome NGS data were concordant with available CR Sanger haplotypes with the exception of three samples (Supplemental Files 1–2). Two of these discordant samples failed to produce usable Sanger sequencing data during initial high-throughput (automated) CR processing [15] and required manual reprocessing. The NGS data for these two samples were reproduced from stock tubes and the mitogenome haplotypes were shown to be consistent across processing events. Thus, the robust QC of these data identified apparent sample switches in the CR Sanger data. The two CR haplotypes were replaced by NGS-derived mitogenome data in EMPOP. The third sample (COAF060) was shown to be a low-level mixture of degraded and intact DNAs; therefore, the previously generated CR haplotype could not be replicated with the two-amplicon long-range mitogenome NGS method. Since the authentic mtDNA haplotype is uncertain for this sample, the CR haplotype was removed from EMPOP and it was not replaced with mitogenome data. CR Sanger data were available for seven other NGS mixed samples (six AGTC-CO and one NIST), and review of these data showed no evidence of mixtures. Though the background noise of Sanger sequencing may mask the low-level (<10%) mixtures identified during NGS processing, it is likely that the contamination occurred after CR Sanger data generation was completed (more than ten years ago) and consequently these seven CR haplotypes were maintained in EMPOP. CR Sanger haplotypes concordant with the NGS data were replaced in EMPOP by the newly generated mitogenome haplotypes (Table S1).

### 3.5. Heteroplasmy

Length heteroplasmy was observed in 1202 (90.6%) individuals (Table S5). Length heteroplasmy was most common in the CR hypervariable (HV) I and II polycytosine stretches, observed in 263 (19.8%) and 1153 (86.9%) individuals, respectively. Additionally, length heteroplasmy in the CR HVIII polycytosine stretch was observed in a total of 105 (7.9%) individuals with 54 (4.1%) individuals exhibiting length heteroplasmy in the 513–524 AC repeat region. Three samples in the COHS (1) and NTHS (2) datasets exhibited length heteroplasmy at both nps 455 and 463. A total of 97 (7.3%) individuals exhibited other coding region/sequence (CDS) length heteroplasmy. Of note, length heteroplasmy at np 8276 was observed in five individuals with T8277C present in every instance. Additionally, 59 (4.4%) individuals had occurrences of length heteroplasmy at np 12,425, none of which resulted in an indel based on the major molecule.

There were 365 (28%) individuals with observed PHPs (Table 5). Of those, 295 individuals (80.8%) had one PHP present and 70 (19.2%) had more than one PHP present, with 60 (16.4%) individuals having two PHPs and ten (2.7%) individuals having three PHPs. Three PHPs was the most observed per individual. A total of 170 PHPs with <10% minor nucleotide frequency were confirmed in at least two separate amplification events. Of the 191 attempted PHP confirmations, 21 (11%) PHPs in 13 samples (12 DoDSR and 1 NIST) could not be confirmed and were not included in the finalized datasets. A majority (17) of the unconfirmed PHPs, all in the DoDSR data, were observed in samples with low amplicon yields (<4 ng/µL) in the original data. Four other PHPs could not be confirmed despite having original amplicon yields > 4 ng/µL. In addition, the original CR Sanger data were utilized for the confirmation of 13 PHPs. In all 13 confirmations, the PHPs were not initially called in the original CR haplotype and the minor bases frequencies were only 7.5% on average. However, upon more targeted review of the data, the 13 PHPs could be observed above background noise in the Sanger sequencing data. It was noted that PHPs with a minor base frequency of at least 5.8% could be retroactively noted in the Sanger sequencing data. In three cases, the PHPs were detected in the NGS data but could not ultimately be observed in the Sanger sequencing data. Every PHP that was originally noted in the Sanger sequencing data was also observed in the NGS data.

**Figure 2:**
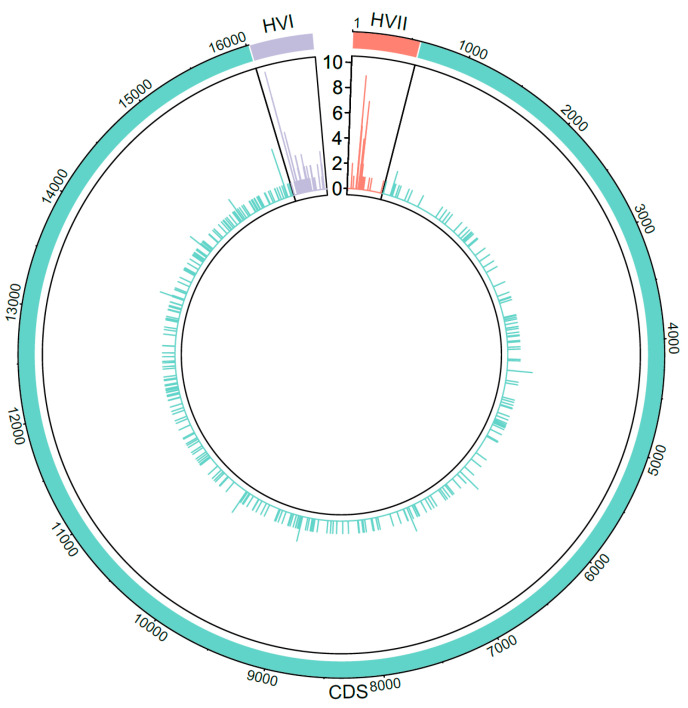
Distribution and count of the 446 observed point heteroplasmies across the mitochondrial genome including the two hypervariable (HVI and HVII) regions of the control region and coding region/sequence (CDS).

A total of 446 PHP variants were observed over 373 unique positions (Supplemental File 2). Of the 446 PHP variants, 149 (33.4%) were located in the CR with multiple occurrences at nps 146 (9), 204 (7), and 16,093 (10) (Figure 2). Only ten positions in the CDS had a PHP variant occur more than once (nps 709, 4384, 6221, 7270, 8987, 9984, 13,477, 14,364, 15,115, 15,924), with np 15,924 having the most at four occurrences. Of the 446 recorded PHPs, 424 (95.1%) were transitions, 228 being pyrimidine transitions (Y) and 196 being purine transitions (R). In the CR, the majority of PHPs (62.4%) were pyrimidine transitions. In contrast, purine and pyrimidine transitions were approximately equal in the CDS with 149 (50.2%) R and 135 (45.4%) Y. Two CR positions had occurrences of both transversion- and transition-type PHPs: np 16192 was observed as C16192Y and C16192S, and np 16,265 was observed as A16265R and A16265M. Additionally, CDS np 6221 was observed as both T6221Y and T6221R in the datasets.

**Figure 3:**
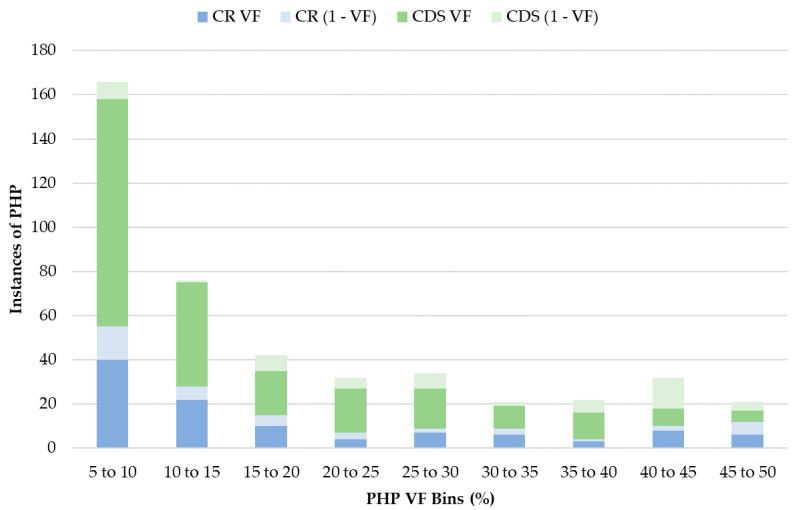
Variant frequencies (VF) of observed point heteroplasmy (PHP) in the control region (CR) (blue) and the coding region/sequence (CDS) (green). For PHPs with VFs higher than 50%, the frequency was subtracted from one (1–VF) and is shaded in light blue or light green.

PHP VF was also examined to assess whether the variant from the rCRS was the major/dominant base (Figure 3). It was found that typically the variant from the rCRS was the minor base, with 143 (32%) of the PHPs having a VF between 5 and 10%. In fact, the percentage of PHPs with a VF below 15% (47.5%) was approximately equal to that with a VF above 15% (52.5%). A larger proportion of the CR PHPs (28.9%) than the CDS PHPs (18.2%) had VFs over 50%.

### 3.6. Population-Level Analyses

Summary statistics were calculated for all datasets (Table 6). When PHPs were included, the highest haplotype diversity was 1 since all haplotypes were unique in the COAF and COCN datasets. The highest observed RMP was in the COHS dataset (1.09%) and the lowest in the NTAF and NTCN datasets (0.41%) when PHPs were included. As expected, the RMPs were lower and the haplotype diversity higher for all datasets when PHPs were included. The lowest haplotype diversity was observed in the COHS dataset regardless of whether PHPs were included or excluded from the pairwise comparisons (0.9983 and 0.9958, respectively). 

**Figure 4:**
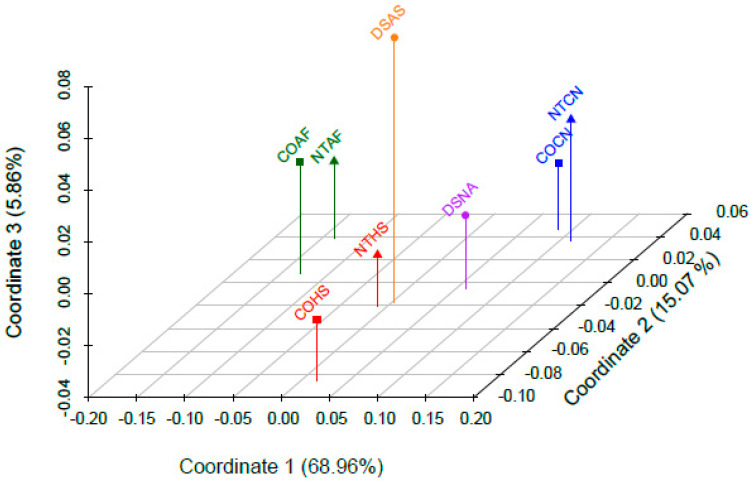
Principal coordinate analyses of the eight datasets. Each metapopulation is labeled in a different color (African American in green, Caucasian in blue, Hispanic in red, Asian American in orange, and Native American in purple). COAF = Colorado African American; COCN = Colorado Caucasian; COHS = Colorado Hispanic; NTAF = National Institute of Standards and Technology (NIST) African American; NTCN = NIST Caucasian; NTHS = NIST Hispanic; DSAS = Department of Defense Serum Repository (DoDSR) Asian American; DSNA = DoDSR Native American.

Genetic distance was evaluated by computing pairwise Fst values for all eight datasets, which are provided in Table S6. The *p*-values were statistically significantly different at a *p* < 0.05 level for all pairwise comparisons except for those between the two Caucasian datasets (COCN and NTCN) and the two African American datasets (COAF and NTAF). Summary statistics combining these datasets are available in Table S7. Conversely, the two Hispanic datasets (COHS and NTHS) were found to be statistically significantly different from each other. Although only represented by a single dataset per metapopulation, the Native American (DSNA) and Asian American (DSAS) datasets were statistically significantly different from all other datasets. As shown in the PCoA plot (Figure 4), approximately 85% of the variation between datasets was represented by coordinates 1 and 2. These first two coordinates (1 and 2) show how closely spaced the two Caucasian and two African American datasets are to each other. The two Hispanic datasets are very close to one another when considering coordinates 1 and 3, but coordinate 2 separates them. It is interesting to note that although coordinate 3 only explains 5.86% of the variation, it distinguishes the NTHS from the DSAS datasets, which are similarly located when considering only coordinates 1 and 2 (Figure S1).

### 3.7. Haplogroup Distribution

**Figure 5:**
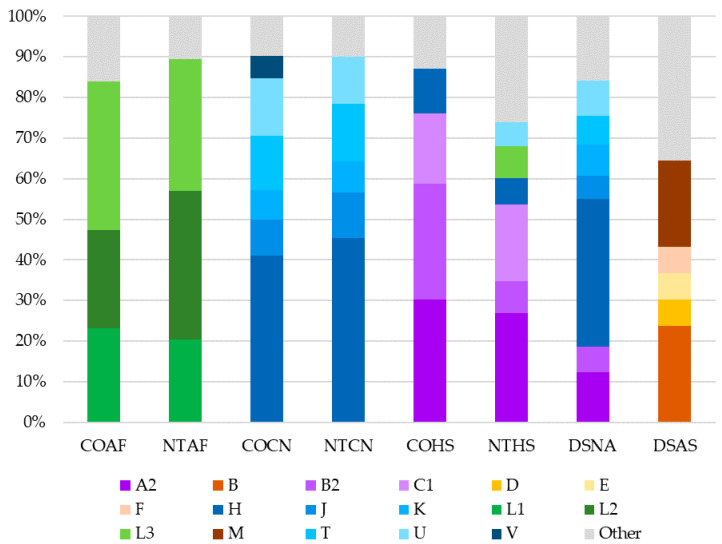
Proportions of haplogroups observed within each dataset. African haplogroups are green, European haplogroups are blue, Native American haplogroups are purple, and Asian haplogroups are orange. Haplogroups representing less than 5% of haplotypes within each dataset have been grouped together and are shaded in grey. For complete haplogroup distribution and frequencies, see Table S8. COAF = Colorado African American; NTAF = National Institute of Standards and Technology (NIST) African American; COCN = Colorado Caucasian; NTCN = NIST Caucasian; COHS = Colorado Hispanic; NTHS = NIST Hispanic; DSNA = Department of Defense Serum Repository (DoDSR) Native American; DSAS = DoDSR Asian American.

**Figure 6:**
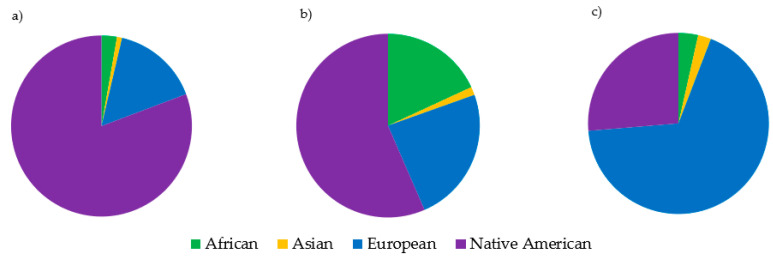
Mitochondrial DNA ancestry proportions in (**a**) the Analytical Genetic Testing Center in Colorado (AGTC-CO) Hispanic (COHS), (**b**) the National Institute of Standards and Technology (NIST) Hispanic (NTHS), and (**c**) the Department of Defense Serum Repository (DoDSR) Native American (DSNA) datasets. Ancestry was classified on a continental level based on the assigned mitochondrial DNA haplogroup.

Haplogroups were assigned to all samples, and haplogroup frequencies were calculated for all datasets (Table S8). African L haplogroups had the highest frequencies in both of the African American datasets, making up 86.6% of the COAF dataset and 93.3% of the NTAF dataset (Figure 5). The Caucasian datasets were comprised mostly of European (H, HV, I, J, K, T, U, V, W, X) haplogroups (97.3% in COCN and 96.2% in NTCN). There was a notable difference between the haplogroup distributions in the two Hispanic datasets (Figure 6a,b). In the NTHS dataset, 18.1% of haplotypes belonged to African haplogroups, whereas only 2.8% of haplotypes in the COHS had African mitochondrial lineages. Furthermore, NTHS had approximately 50% more European haplogroups than COHS. Within the Native American haplogroups that comprised the highest proportion in both NTHS and COHS, the frequency of Native American haplogroup B2 differed between the datasets. The NTHS had 8.0% B2, yet more than one-quarter (28.4%) of the COHS haplotypes belonged to B2. In the DSAS dataset, Asian haplogroups B and M had the highest frequencies at 23.7% and 21.3%, respectively. In the DSNA dataset, European haplogroup H was most frequent at 36.3% (Figure 6c). In fact, 67.8% of haplotypes in the DSNA dataset were assigned to haplogroups of European ancestry, with only 26.3% of haplotypes associated with Native American haplogroups (A2 12.3%, B2 6.4%, C1 3.5%, and C4c 1.2%). Sunburst plots for each dataset are given in Figure S2.

## 4. Discussion

Forensic-quality mitogenomes were generated for 97% (1327/1365) of the samples attempted in this study. Despite slight differences in NGS processing (Table 2), no loss of quality was observed in the data from any one method. Though the read count and read depth metrics differ between each data source, these metrics can be affected by a variety of factors such as the number of samples included in the MiSeq run, the read type (paired end v. single end), and the cluster density of the run. The major base frequency metrics, which are more indicative of sample data quality, were comparable between all three data sources, averaging > 99% when heteroplasmy was excluded. This indicates that minimal background noise (<1%) was observed in all datasets regardless of variations in sample type and processing. Thus, the two-amplicon long-range approach (supplemented with four-amplicon enrichment for lower quality samples), HyperPlus library preparation, and MiSeq sequencing, combined with the robust analysis pipeline, produced more than 1300 platinum-quality mitogenomes.

The four-amplicon enrichment approach was useful for processing samples that previously failed with the two-amplicon approach. However, NUMT interference was observed at 2–3% minor base frequency in the 4C amplicon of three samples. These NUMTs were below the 5% minor variant detection but emphasized the importance of data QC when analyzing at lower variant detection thresholds (<5%). It is also noteworthy that all three NUMTs were observed in the NIST samples extracted from whole blood, which has a higher likelihood for co-enrichment of NUMTs [49]. However, no NUMTs were observed in data generated with the two-amplicon method, demonstrating the advantage of using the two-amplicon enrichment approach to generate reliable mitogenome data. The application of two-amplicon long-range enrichment allows primers to be specific for the mitogenome, opposed to small-amplicon approaches that may be homologous to regions of the nuclear genome due to primer design restrictions and mtDNA variation [5,11,22,50].

STR testing on samples with shared haplotypes was an important aspect of QC in this study, helping to identify duplicate or related samples for removal from the database. The inclusion of maternally related individuals in mtDNA databases can result in inaccurate estimation of haplotype frequencies [37]. The use of both STR and SNP data allowed for the identification of two sets of more distant (second- and third-degree) relatives compared to traditional STR testing, which is typically only suitable for kinship analysis of first-degree relatives [51,52]. Since these more distant relatives were numbered sequentially in each case, it was presumed that the relatives were sampled simultaneously and that a genetic relationship was recognized between them. Other cases in which genetic relatives, recognized or not, were randomly sampled on separate occasions, would be better representative of the general population and might not be excluded from a mitogenome database. As new STR and SNP assays allow for more distant kinship testing [53], it will be important to develop guidelines for the appropriate threshold for inclusion of relatives in population databases of lineage markers.

The automated nature of NGS data analysis combined with read count confirmation ensured that the major molecule was accurately assigned in homopolymer regions for all samples, in accordance with ISFG guidelines for population data [14]. The robust analysis workflow used in this study identified haplotypes with indels in common length heteroplasmy regions, which are recognized in EMPOP and can be disregarded in EMPOP haplotype queries. Indels were furthermore observed at eight additional CR and CDS sites that are not currently possible to ignore in EMPOP queries. However, these eight additional indels were observed at low frequency (<0.1% of haplotypes) with only one (nps 1878, 2141, 2417, 8289, 14,529, 15,545, 16,166, 16,296) or two (np 71) samples each. Because of their rarity, indels in these eight regions may not require special handling in database queries.

The authenticity of low-frequency PHPs presented in this study is ensured by the fact that every PHP with a minor base frequency < 10%, down to the minimum detection threshold of 5%, was confirmed in at least two separate amplification events. The importance of PHP confirmation for QC was emphasized by the fact that 22 PHPs could not be reproduced in the second amplification. Often in these cases, re-amplification would result in higher amplicon yields, but the PHP was not observed, indicating that the original observation was due to stochastic error. Inclusion of these spurious PHPs in the final datasets would over-represent mtDNA heteroplasmy rates and thus inflate haplotype diversity estimations [54,55,56]. Of the reported PHPs, the majority (66.6%) were located in the CDS, consistent with the findings of [57] in which 61.4% of observed PHPs were located in the CDS. In this study, PHPs were most frequently observed at CR positions 146, 152, 204, and 16,093, all of which are known PHP hotspots [57,58,59,60]. Unlike the findings in [57,58], not all CDS PHPs observed in this study were unique, with ten positions having more than one PHP occurrence. However, this difference is likely due to the larger sample size of the present study, which is one of the largest forensic mitogenome datasets published to date. Additionally, these previous studies utilized Sanger sequencing and thus were limited to a 10–20% threshold, the point at which when minor base signal could be confidently discerned from CE background noise. This is in contrast to the 5% heteroplasmy detection threshold used in this study, which can reveal lower-level variants than Sanger sequencing. Finally, the majority (95.1%) of the reported PHPs were transition-type, consistent with findings in [57], in which 96% of PHPs were transitions. 

When pairwise Fst values were used to estimate genetic distance between the eight datasets, it was found that all comparisons except for those between the two African American (COAF and NTAF) and two Caucasian (COCN and NTCN) datasets were statistically significantly different from one another. Based on these findings, it would be reasonable for a laboratory to combine the African American and Caucasian datasets to increase sample size when conducting haplotype match statistics. Conversely, the difference between the two Hispanic (COHS and NTHS) datasets was noteworthy. Though the largest proportions of haplogroups for both the COHS and NTHS Hispanic datasets were of Native American ancestry (80.7% and 56.5%, respectively), the NTHS had a much larger proportion of European and African lineages (42% total) compared to the COHS dataset (18.3% total). The difference in haplogroup distribution between these two Hispanic datasets is likely due to the differing geographic origin of each sample set, as the NTHS samples were collected in the northeast and southern United States whereas the COHS samples were mostly from Colorado. Based on the 2010 U.S. Census, Hispanics from the western United States (including Colorado) are predominantly from Mexico and Central America, whereas a large proportion of Hispanics in the northeast and Florida originate from the Caribbean [61]. As a result, the differing population structure of the Central American and Caribbean parental populations likely contributed to the observed differences between the two Hispanic datasets. In particular, the greater proportion of African ancestry in Caribbean populations [62] may explain the prevalence of African lineages in the NTHS dataset [25]. The geographic sampling differences between the Hispanic datasets is furthermore evident in their haplogroup B2 proportions. Haplogroup B2 is a Native American lineage found in relatively high frequencies in the southwestern U.S. [63,64,65]. Therefore, the proportion of B2 haplotypes in the COHS dataset, which includes individuals primarily from Colorado (a southwestern state), was greater than that of the NTHS dataset originating from eastern and southern states. These analyses highlight the distinctiveness of the two geographically disparate Hispanic mitogenome datasets included in this study.

It was not unexpected that the Asian (DSAS) and Native American (DSNA) datasets were different from the other datasets (and each other), given that there was only one dataset per metapopulation. The mtDNA haplogroup distribution observed in the Native American (DSNA) dataset was interesting, with 67% of haplotypes belonging to European haplogroups. This finding explains why the Native American dataset plotted between the Caucasian and Hispanic datasets, considering the high proportion of Native American haplotypes in the Hispanic datasets. The finding that the Native American dataset contained a lower proportion of Native American haplotypes than the Hispanic datasets underscores the complexity behind self-identification as it relates to DNA and genetic ancestry. This demonstrates that mitochondrial ancestry may be inconsistent with expectations based on the self-reported metapopulation, especially for admixed and indigenous groups.

## 5. Conclusions

In this study, platinum-quality mitogenome reference data were reported for 1327 individuals representing five U.S. metapopulations: African American, Caucasian, Hispanic, Asian American, and Native American. These haplotypes were generated using robust processing and analysis methods with a rigorous QC procedure. The use of two-amplicon long-range enrichment minimized NUMT co-amplification and allowed for the detection of low-level variants down to 5% minor base frequency. An automated data analysis method combined with a dual-analyst review minimized the bias from data interpretation and assisted with the detection of errors. Furthermore, a rigorous QC procedure ensured the authenticity of reported heteroplasmy, demonstrated concordance with previous CR Sanger data, and supported the removal of duplicate or related samples from the database. These platinum-quality haplotypes will augment the forensic-quality EMPOP database, allowing for more accurate estimations of mitogenome haplotype frequencies. These data will also be useful for downstream analyses of mtDNA substitution rates and heteroplasmy trends.

## Figures and Tables

**Table 1:** Sample set information for all 1363 samples.

Sample Set	Source	U.S. Geographic Origin	Metapopulation	Sample Type	Count
COAF	Analytical Genetic Testing Center (Denver, CO)	Colorado *	African American	Whole blood, buccal swabs	123
COCN	Analytical Genetic Testing Center (Denver, CO)	Colorado *	Caucasian	Whole blood, buccal swabs	118
COHS	Analytical Genetic Testing Center (Denver, CO)	Colorado *	Hispanic	Whole blood, buccal swabs	113
NTAF	National Institute of Standards and Technology (Gaithersburg, MD)	Multiple States	African American	Whole blood	258
NTCN	National Institute of Standards and Technology (Gaithersburg, MD)	Multiple States	Caucasian	Whole blood	262
NTHS	National Institute of Standards and Technology (Gaithersburg, MD)	Multiple States	Hispanic	Whole blood	139
DSAS	Department of Defense Serum Repository (Silver Spring, CO)	Multiple States/Territories	Asian American	Serum	175
DSNA	Department of Defense Serum Repository (Silver Spring, CO)	Multiple States	Native American	Serum	175

COAF = Colorado African American; COCN = Colorado Caucasian; COHS = Colorado Hispanic; NTAF = National Institute of Standards and Technology (NIST) African American; NTCN = NIST Caucasian; NTHS = NIST Hispanic; DSAS = Department of Defense Serum Repository (DoDSR) Asian American; DSNA = DoDSR Native American. * The majority of samples were collected from individuals living in Colorado, though some samples in the Analytical Genetic Testing Center sets may have originated from other U.S. states.

**Table 2:** Summary of laboratory processing methods for each sample source: the Analytical Genetic Testing Center in Colorado (AGTC-CO), the National Institute of Standards and Technology (NIST), and the Department of Defense Serum Repository (DoDSR). Samples were processed either at the Armed Forces Medical Examiner System’s Armed Forces DNA Identification Laboratory (AFMES-AFDIL) or at the Applied Genetics laboratory at NIST.

Sample Source	Processing Laboratory	Amplification Input (µL)	Amplicon Purification	Library Preparation			Sequencing		
				Input (ng)	Reaction	Method	Input (pM)	Reagent Kit	Read Type
AGTC-CO	AFMES-AFDIL	3	Yes	150	Half-reaction	Manual	12	150 cycle v3	Single end
NIST	NIST	2	No	350	Full-reaction	Manual	20	600 cycle v3	Paired end
DoDSR	AFMES-AFDIL	5	Yes	50	Half-reaction	Automated	12	600 cycle v3	Paired end

**Table 3:** Breakdown of sample success rate by dataset.

Dataset	Samples Attempted	Finalized Samples	Passing		Excluded			
			Two Amplicon	Four Amplicon	Failed	Mixed	Duplicate	Related
COAF	123	112	112	0	6	3	2	0
COCN	118	112	112	0	5	1	0	0
COHS	113	109	109	0	1	3	0	0
NTAF	258	256	251	5	1	1	0	0
NTCN	262	260	258	2	1	0	0	1
NTHS	139	138	138	0	0	0	0	1
DSAS	175	169	165	4	3	3	0	0
DSNA	175	171	158	13	1	3	0	0
Total	1363	1327	1303	24	18	14	2	2

COAF = Colorado African American; COCN = Colorado Caucasian; COHS = Colorado Hispanic; NTAF = National Institute of Standards and Technology (NIST) African American; NTCN = NIST Caucasian; NTHS = NIST Hispanic; DSAS = Department of Defense Serum Repository (DoDSR) Asian American; DSNA = DoDSR Native American.

**Table 4:** Analysis metrics for each data source. The metric values from each set were averaged.

Data Source	Total Reads	Reads After Trim	Reads Mapped	Trimmed Reads Mapped (%)	Average Read Depth	Average Major Base Frequency (%)	Average Major Base Frequency Excluding Heteroplasmy (%)	Average Variant Position Read Depth
AGTC-CO	352,136	313,022	293,667	94	1658.8	98.6	99.5	1499.6
NIST	383,834	272,090	256,309	95	1558.4	98.0	99.1	1466.0
DoDSR	688,530	566,775	504,600	90	2385.4	97.5	99.2	2198.7

AGTC-CO = Analytical Genetic Testing Center in Colorado; NIST = National Institute of Standards and Technology; DoDSR = Department of Defense Serum Repository.

**Table 5:** Number of individuals with observed point heteroplasmies (PHPs) in each dataset.

Dataset	Total Individuals	Total PHPs	Individuals with PHPs	Individuals with 1 PHP	Individuals with 2 PHPs	Individuals with 3 PHPs
COAF	112	37	31 (28%)	26	4	1
COCN	112	41	30 (27%)	20	9	1
COHS	109	36	27 (25%)	20	5	2
NTAF	256	77	60 (23%)	43	17	0
NTCN	260	92	77 (30%)	65	10	2
NTHS	138	53	43 (31%)	34	8	1
DSAS	169	62	54 (32%)	49	2	3
DSNA	171	48	43 (25%)	38	5	0
All	1327	446	365 (28%)	295	60	10

COAF = Colorado African American; COCN = Colorado Caucasian; COHS = Colorado Hispanic; NTAF = National Institute of Standards and Technology (NIST) African American; NTCN = NIST Caucasian; NTHS = NIST Hispanic; DSAS = Department of Defense Serum Repository (DoDSR) Asian American; DSNA = DoDSR Native American.

**Table 6:** Summary statistics. Observed and empirical random match probabilities (RMPs) and haplotype diversity were calculated for the entire mitogenome when point heteroplasmy (PHP) were included and excluded. Length heteroplasmy nps 16,193, 309, 315, 455, 463, 573, 960, 5899, 8276, and 8285 were ignored for these calculations.

Dataset	Sample Size	Including PHP					Excluding PHP				
		Total Haplotypes	Unique Haplotypes	Observed RMP (%)	Empirical RMP (%)	Haplotype Diversity	Total Haplotypes	Unique Haplotypes	Observed RMP (%)	Empirical RMP (%)	Haplotype Diversity
COAF	112	112	112	0.89	0.00	1	110	108	0.92	0.03	0.9997
COCN	112	112	112	0.89	0.00	1	112	112	0.89	0.00	1
COHS	109	102	97	1.09	0.17	0.9983	94	83	1.34	0.42	0.9958
NTAF	256	251	247	0.41	0.02	0.9998	246	237	0.42	0.03	0.9997
NTCN	260	254	250	0.41	0.02	0.9998	250	244	0.43	0.05	0.9995
NTHS	138	131	127	0.86	0.14	0.9986	125	116	0.97	0.24	0.9976
DSAS	169	167	165	0.61	0.01	0.9999	165	161	0.62	0.03	0.9997
DSNA	171	167	163	0.61	0.03	0.9997	164	159	0.65	0.07	0.9993

COAF = Colorado African American; COCN = Colorado Caucasian; COHS = Colorado Hispanic; NTAF = National Institute of Standards and Technology (NIST) African American; NTCN = NIST Caucasian; NTHS = NIST Hispanic; DSAS = Department of Defense Serum Repository (DoDSR) Asian American; DSNA = DoDSR Native American.

## Supplementary Materials

The following are available online at https://www.mdpi.com/2073-4425/11/11/1290/s1, Table S1: EMPOP and GenBank accession numbers. Table S2: Long-range two-amplicon (2-amp) and four-amplicon (4-amp) sets, primers and sequences. Table S3: Analysis metrics for each dataset. Table S4: Kinship analysis results for the two sets of NIST samples in which one sample in the set was removed due to distant relatedness. Table S5: Observations of length heteroplasmy across the mitochondrial genome for each dataset. Table S6: Pairwise Fst values and associated p-values. Table S7: Summary statistics for combined African American datasets and combined Caucasian datasets. Table S8: Frequencies of observed haplogroups within each dataset. Figure S1: Two-dimensional principal coordinate analysis plots produced from pairwise Fst values. Figure S2: Mitochondrial ancestry proportions by dataset. Supplemental File 1: Mitochondrial DNA control region haplotypes previously generated with Sanger sequencing. Supplemental File 2: Custom analysis workflows developed in CLC Genomics Workbench for the analysis of next-generation sequencing mitochondrial genome data. Supplemental File 3: Mitochondrial genome haplotypes generated with a robust next-generation sequencing method. Supplemental File 4: Consensus sequences.
