# Supplementary material for: Platinum-Quality Mitogenome Haplotypes from United States Populations
Source: Genes (Basel). 2020 Oct 29;11(11):1290. doi: 10.3390/genes11111290 (PMC7716222; doi:10.3390/genes11111290)
Supplement: Supplementary file 1 [file genes-11-01290-s001.zip › Supplementary Figures.docx]

Figure S1. Two-dimensional principal coordinate analysis plots produced from pairwise Fst values. The plots show (a) coordinates 1 and 2 and (b) coordinates 1 and 3. COAF = Colorado African American; COCN = Colorado Caucasian; COHS = Colorado Hispanic; NTAF = National Institute of Standards and Technology (NIST) African American; NTCN = NIST Caucasian; NTHS = NIST Hispanic; DSAS = Department of Defense Serum Repository (DoDSR) Asian American; DSNA = DoDSR Native American.


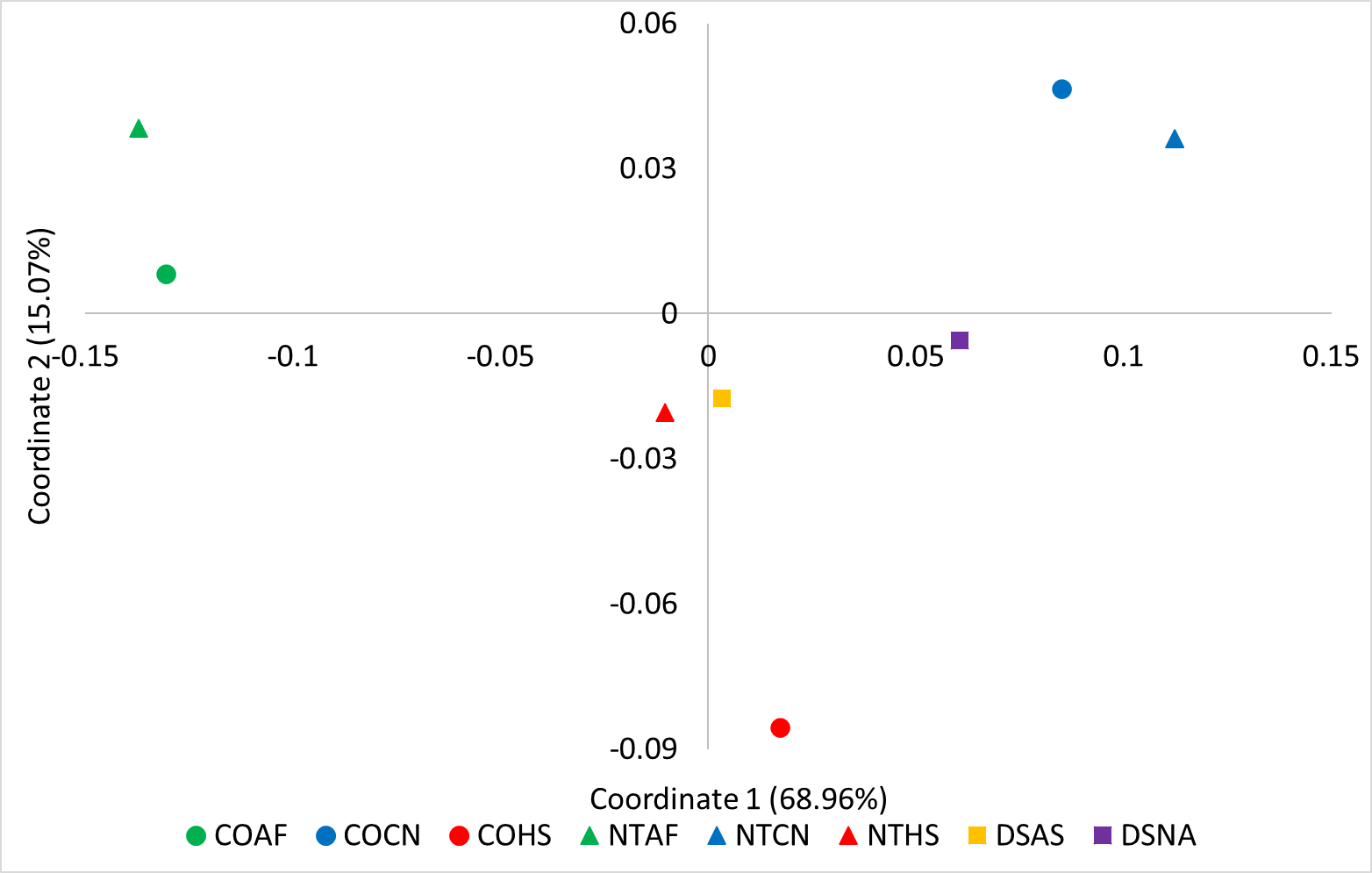


a) Coordinates 1 and 2


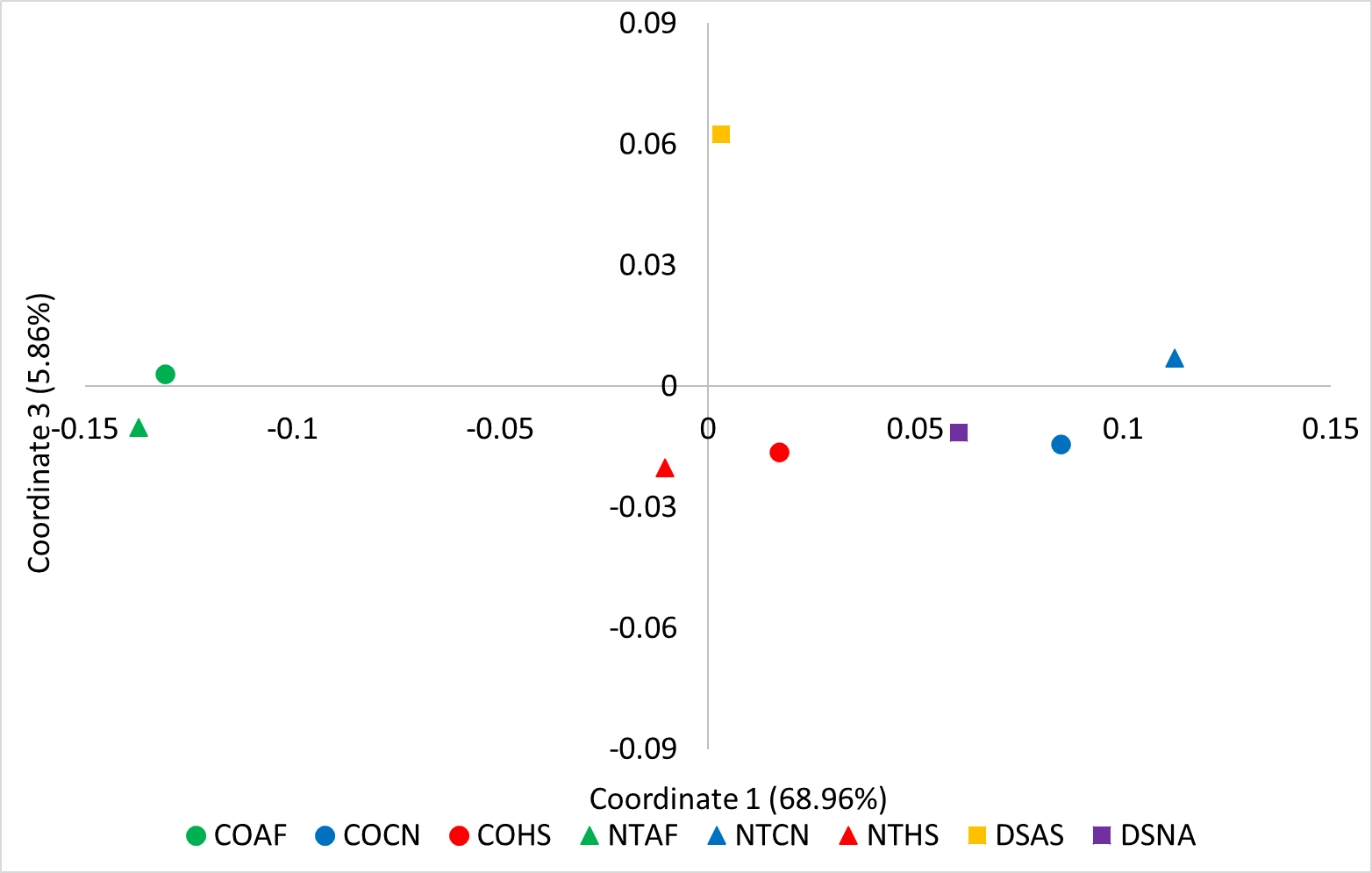


b) Coordinates 1 and 3

Figure S2. Mitochondrial ancestry proportions by dataset: a) COAF, b) NTAF, c) COCN, d) NTCN, e) COHS, f) NTHS, g) DSAS, and h) DSNA. Mitochondrial ancestries were classified on a continental level based on the assigned mitochondrial DNA haplogroup: African (green), Asian (yellow), European (blue) and Native American (purple). COAF = Colorado African American; COCN = Colorado Caucasian; COHS = Colorado Hispanic; NTAF = National Institute of Standards and Technology (NIST) African American; NTCN = NIST Caucasian; NTHS = NIST Hispanic; DSAS = Department of Defense Serum Repository (DoDSR) Asian American; DSNA = DoDSR Native American.


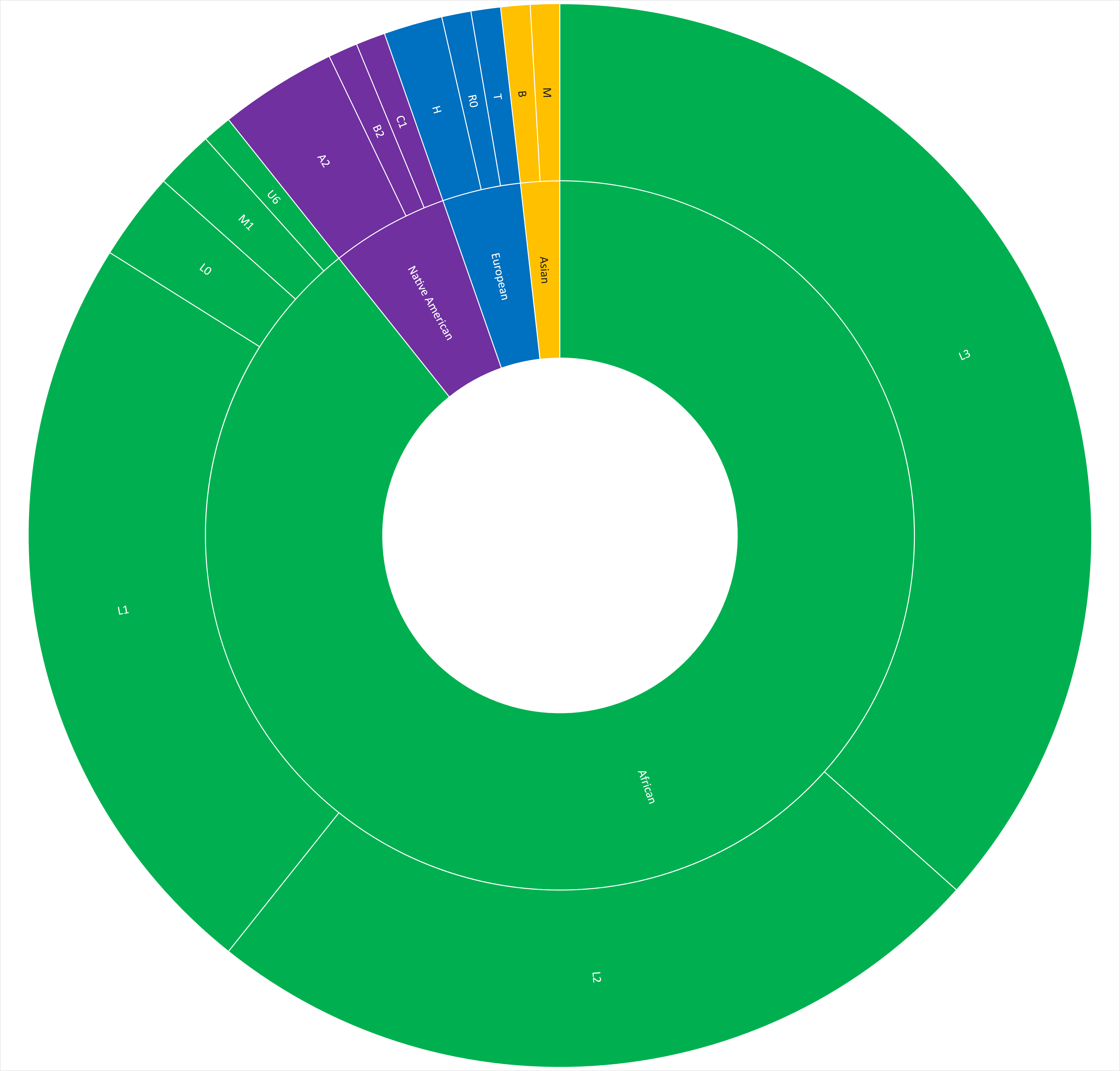


a) COAF


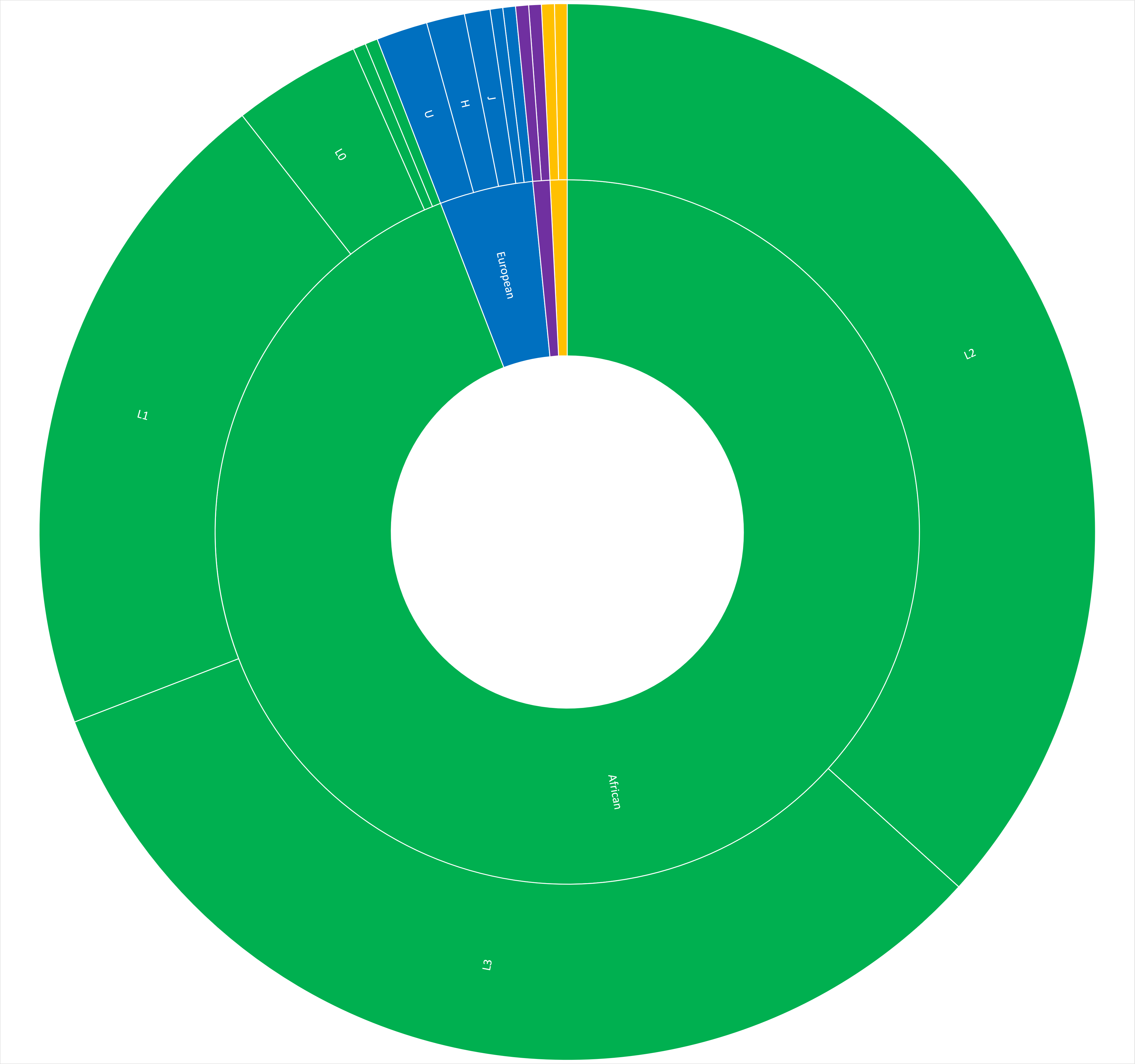


b) NTAF


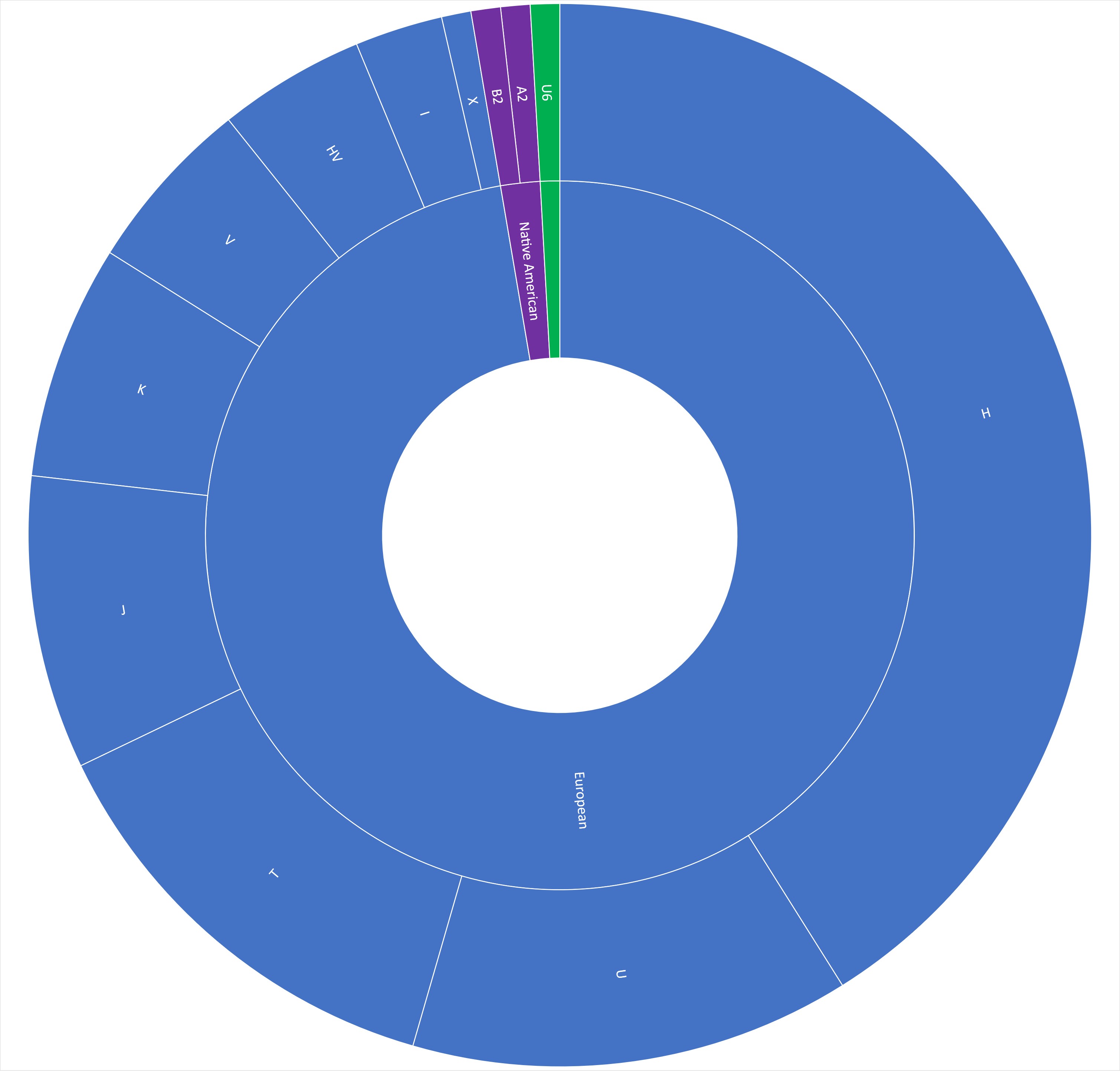
c) COCN


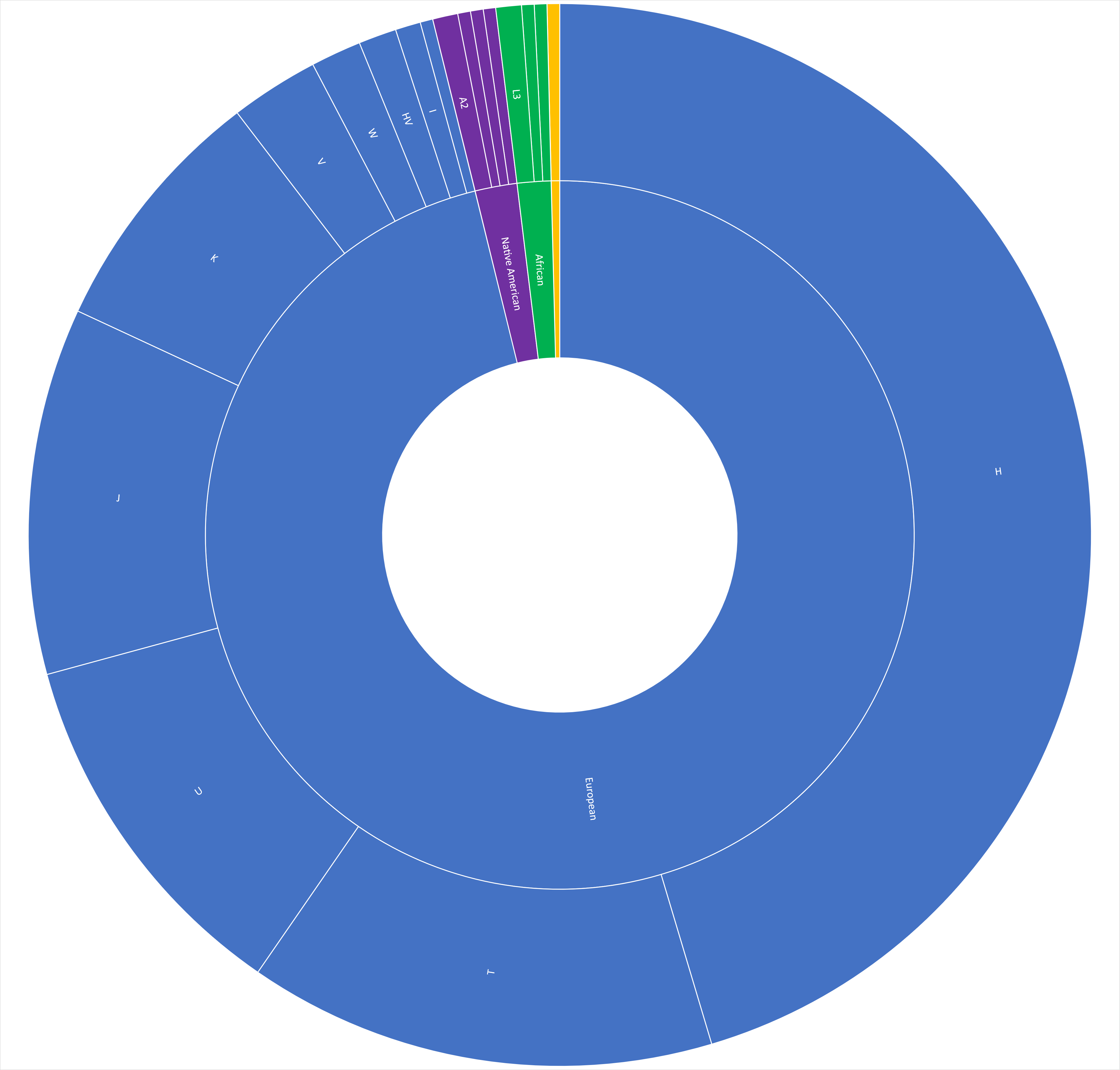


d) NTCN


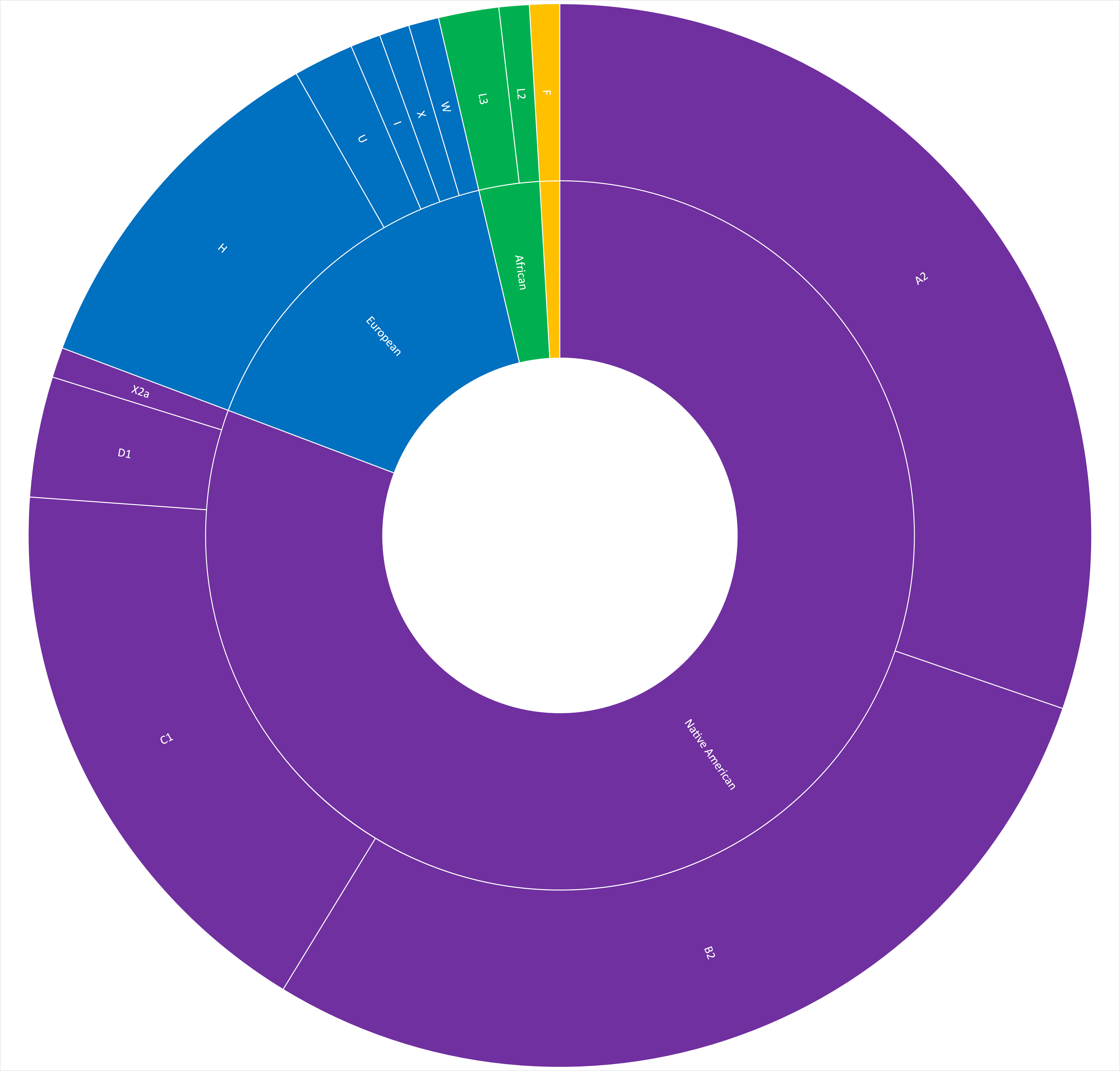


e) COHS


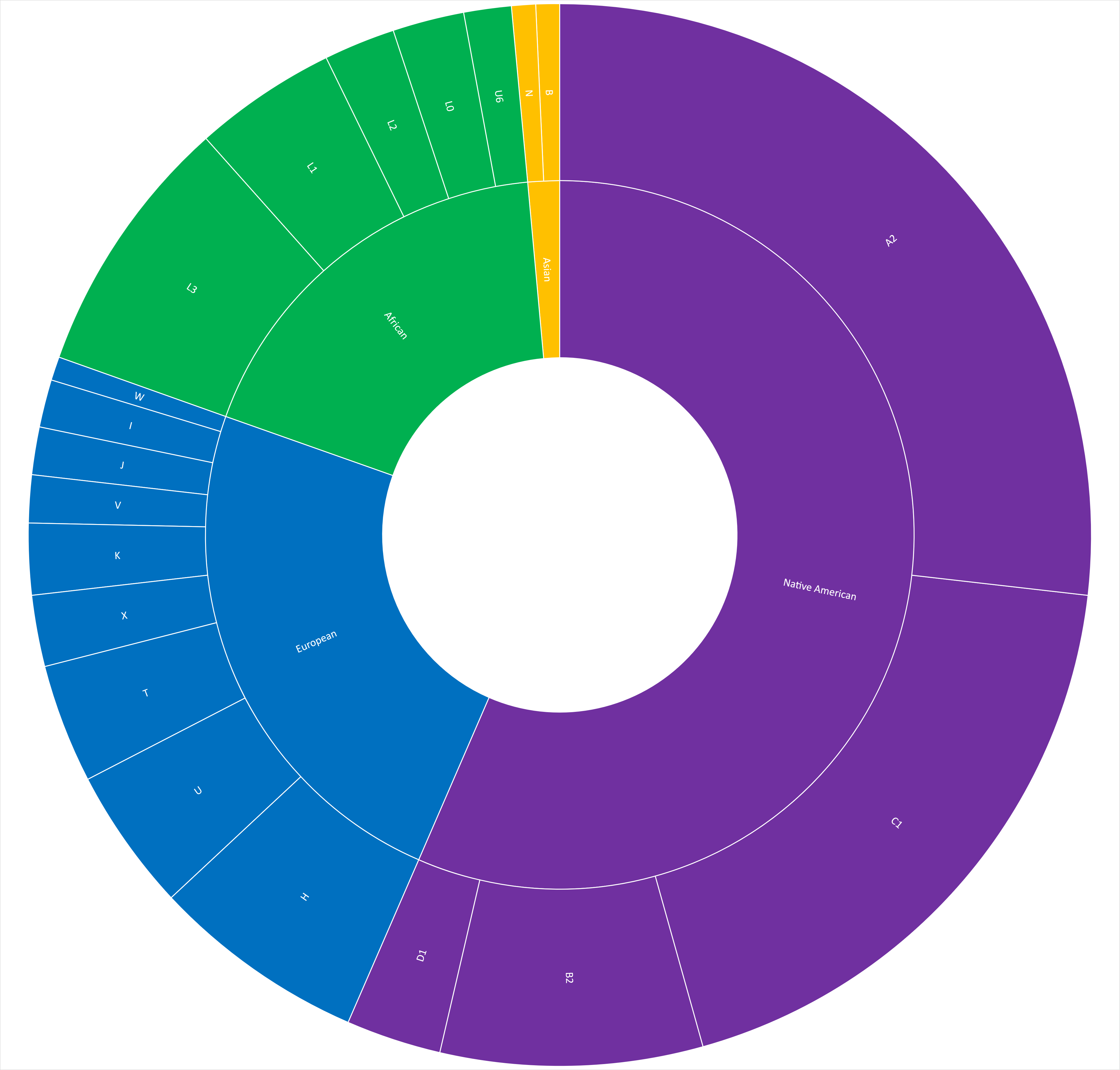


f) NTHS


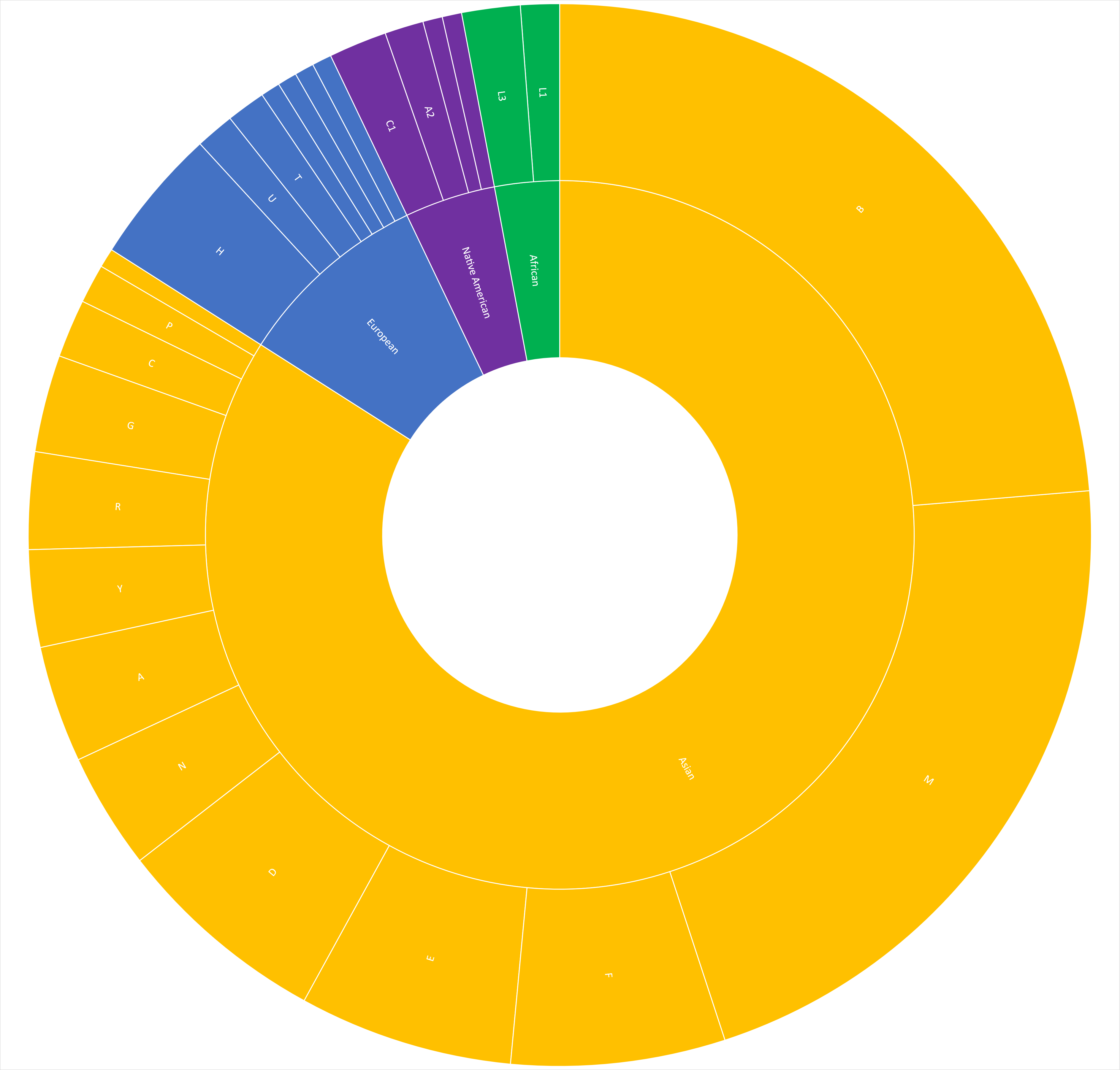


g) DSAS


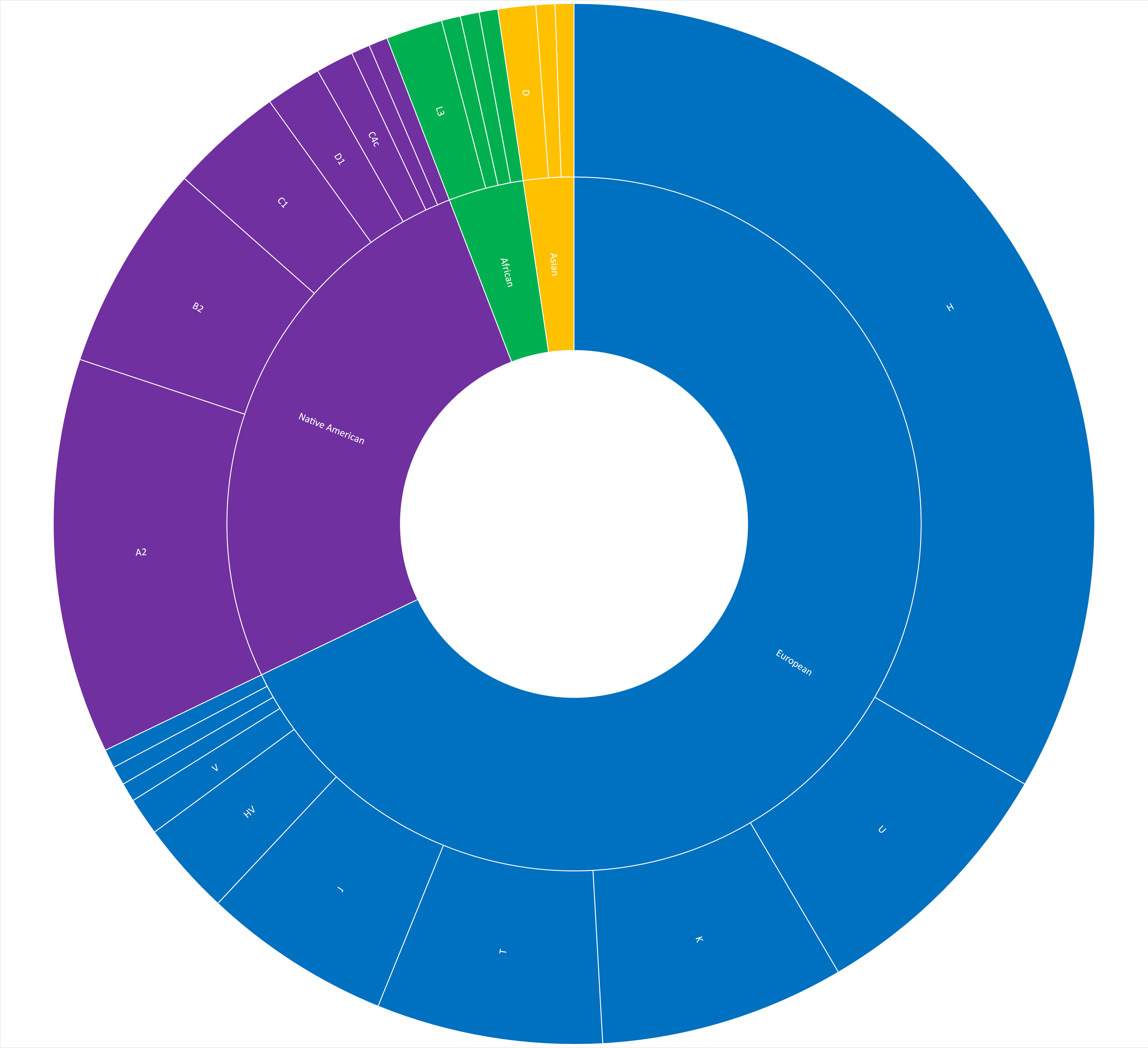


h) DSNA
